# Parasitic Fauna of Free‐Living *Chelonoidis denticulatus* From the Rio Acre Ecological Station and the Municipality of Cruzeiro do Sul, Western Amazon, Brazil

**DOI:** 10.1155/vmi/6647778

**Published:** 2026-01-31

**Authors:** Ester Nascimento da Costa, Caio Bonfanti Gomes, Rayná Girard Madeira, João José de Souza Moura, Muriele Furtado de Assis, Ana Paula Carvalho Gomes, Victória Luiza de Barros Silva, Iago de Sá Moraes, Reiner Silveira de Moraes, Richard de Campos Pacheco, Dirceu Guilherme de Souza Ramos, Francisco Glauco de Araújo Santos

**Affiliations:** ^1^ Laboratory of Pathology and Wildlife, Federal University of Acre, Rio Branco, Acre, Brazil, ufac.br; ^2^ Rio Acre Ecological Station, Chico Mendes Institute for Biodiversity Conservation, Assis Brasil, Acre, Brazil; ^3^ Laboratory of Veterinary Parasitology, Federal University of Jataí, Jataí, Goiás, Brazil; ^4^ Laboratory of Parasitology and Parasitic Diseases, Federal University of Mato Grosso, Cuiabá, Mato Grosso, Brazil, ufmt.br; ^5^ Department of Veterinary Clinical Medicine, São Paulo State University, Botucatu, São Paulo, Brazil, unesp.br

**Keywords:** consumption, one health, parasites, tortoises, wild animals

## Abstract

Tortoises, such as *Chelonoidis denticulatu*s, are described as hosts of many parasites, such as helminths and ticks of the genus *Amblyomma*, which are important vectors of rickettsial infection in Brazil. Additionally, the high consumption of meat from these animals in Acre results in a high risk of zoonotic outbreaks due to contact with the hosts and, consequently, associated pathogens. This study aimed to describe the parasitic fauna of *C. denticulatus.* Two sampling efforts were conducted: the capture of a sample population of tortoises at the Rio Acre Ecological Station (active search) and the collection of viscera from animals consumed in the municipality of Cruzeiro do Sul. Following active search and incidental findings, ectoparasite searches were performed, and fecal samples were collected for coproparasitological examination. After these procedures, the animals were released back into their habitat. In total, seven animals were collected, with ticks on various parts of their bodies. A total of 51 samples, identified as *Amblyomma humerale*, were sent for molecular analysis to search for *Rickettsia* (all negative). Eggs of helminths and protozoan cysts, such as *Entamoeba* spp., were found, indicating environmental contamination and a potential zoonotic risk. Viscera of 10 tortoises from Cruzeiro do Sul were analyzed to search for helminths, and the species *Labiduris zschokkei*, *Chapiniella variabilis*, and *Haltrema* spp. were observed. Considering that the sampled animals were free‐living, the analysis highlights the importance of maintaining environmental quality. With respect to cultural aspects, the consumption of wild animals in the western Amazon of Brazil is evident, and *C. denticulatus* is one of the most consumed species. The contact of these species with humans, in a consumption relationship, is considered a risk factor for the emergence of spillover events, and monitoring the pathogens associated with these species is crucial.

## 1. Introduction

Parasite–host interactions in Amazonian wildlife are still poorly understood, even though they play a key role in the maintenance and transmission of pathogens that may affect both animals and humans [1]. In regions of the western Amazon, where hunting and the consumption of *Chelonoidis denticulatus* are culturally rooted and widely practiced, understanding the parasites associated with this species becomes even more relevant [2].

Reptiles and amphibians are important hosts of several parasites, including mites and ticks [3]. Ticks are of great epidemiological importance, as they are the second largest vector of pathogens, causing significant harm to human and animal health [4]. Ticks of the genus *Amblyomma* are the most aggressive ticks for humans who have contact with wildlife and some domestic animals [5] and are able to transmit rickettsial diseases, such as *Rickettsia amblyommatis*, which is related to *Amblyomma humerale* [6, 7].

*A. humerale* is considered endemic to South America and has been described in many Brazilian biomes, such as the Amazon [8], Cerrado [9], and Atlantic Forest [10]. The immature stages of *A. humerale* can be found in a greater diversity of hosts and can feed on reptiles, birds, and mammals; however, reptiles are also important hosts for the adult phase [11], predominantly *C. denticulatus* (yellow‐footed tortoise) and *Chelonoidis carbonaria* (red‐footed tortoise) [8]. Males are usually found in clusters located on the ventral part of the carapace, whereas females are found on the skin of the head, neck, and pelvic limbs, with a predominance of males over females being common [8, 10].

*C. denticulatus* is found in the Amazon, including Acre, and it is the species of tortoise most consumed in the municipality of Cruzeiro do Sul [12]. This species is also reported to be parasitized by nematodes of the families Ascarididae, Strongylidae, and Atractidae in Brazil. Some examples of species from the first family are *Angusticaecum brevisculum*, from the second are *Chapiniella variabilis* and *Sauricola sauricola,* and from the last are *Klossinemella travassosi*, *Labiduris gulosa*, *Labiduris irineuta*, and *Labiduris zschokkei*, which are all reported in reptiles [13].

The consumption of game fauna is deeply rooted in the region’s culture and has also been increasing in urban areas. This trend poses significant health risks, as the interaction of humans with wildlife puts humans in direct contact with numerous pathogens, especially bodily fluids and feces, as well as handling, preparation, and consumption of bushmeat [1, 2].

This study aimed to describe the intensity, abundance, and parasitic occurrence of *C. denticulatus* in free‐living individuals from the Rio Acre Ecological Station and in the viscera of *C. denticulatus* consumed in the municipality of Cruzeiro do Sul, Acre, in the western Amazon.

## 2. Materials and Methods

### 2.1. Collection Sites and Animal Sampling

**FIGURE 1:**
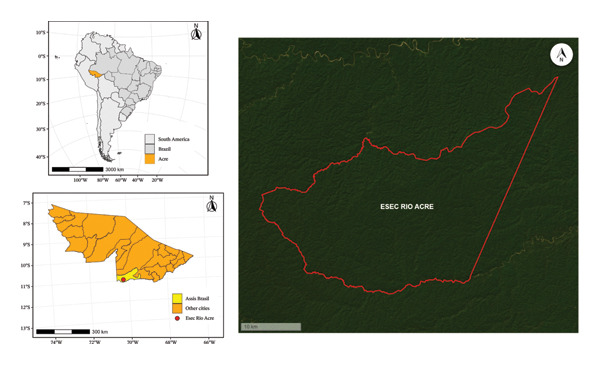
Map of the Rio Acre Ecological Station where free‐living *Chelonoidis denticulatus* were captured for parasitizing analysis.

The study was conducted at the Rio Acre Ecological Station (Figure 1), which is a fully protected conservation unit covering an area of 77,500 hectares with a perimeter of 146,130 m. It is located in the northern region of Brazil, in the southeastern part of the state of Acre, at one of the triborder areas between Brazil, Bolivia, and Peru. The viscera were collected from Cruzeiro do Sul, located 636 km away from Rio Branco, the capital of Acre, which has approximately 89,072 inhabitants, according to the 2020 census.

We sought to capture free‐living *C. denticulatus* specimens through pitfall traps baited as described by Cecchin and Martins [14], with modifications by Vogt [15], and through active search and occasional encounters at the Estação Ecológica do Rio Acre. To avoid the risk of recapturing the same animals, they were marked with nontoxic and waterproof paint, as suggested by Balestra et al. [16]. The captures were carried out according to the sampling hours (144 h), and seven yellow‐footed tortoise samples were collected.

We also published on social media, seeking donors of *C. denticulatus* viscera consumed by the population of Cruzeiro do Sul during the end‐of‐year festivities. An anonymous donation of 10 viscera was made, which were stored frozen for later sample processing.

The present study was registered with the Biodiversity Authorization and Information System (SISBIO), under registration number 88210‐2, and authorized by the Animal Ethics Committee (CEUA) of the Federal University of Acre (UFAC), under license number 11/2023.

### 2.2. Ectoparasites and Rickettsial Analyses

All the captured free‐living animals were physically and visually inspected for the presence of ectoparasites. For the preservation of the ticks, 70% ethanol was used. The ticks were identified to the lowest possible taxonomic level on the basis of morphological data via the identification key of Barros‐Battesti et al. [17].

The ticks were also subjected to molecular tests for *Rickettsia*. DNA was extracted from the identified ticks via guanidine isothiocyanate, as previously described by Sangioni et al. [18]. DNA extractions were screened for *Rickettsia* agents via polymerase chain reaction (PCR) using the primers CS‐78 and CS‐323, which amplify an ∼401‐base pair (bp) fragment of the citrate synthase (*gltA*) gene common to all *Rickettsia* species [19].

Although *A. humerale* is not a confirmed vector of rickettsial pathogens, molecular surveys in the Amazon region have already detected *Rickettsia* DNA in specimens of this species, which justifies the inclusion of a rickettsial screening component in ecological and parasitological surveys involving wildlife.

### 2.3. Coproparasitological Techniques

Feces were collected from all the free‐living *C. denticulatus* specimens and preserved in Merthiolate (or Mercurochrome), iodine, or formalin (MIF) for future coproparasitological analysis through the Willis and spontaneous sedimentation techniques [20].

### 2.4. Helminth Collection and Identification

The analysis of the viscera content was performed under a stereoscopic microscope. Because the viscera obtained from subsistence consumption were received fragmented and mixed, it was not possible to reliably determine the exact organ of origin for helminth infections. Therefore, helminths were analyzed only for presence and abundance. Helminths were stored in 70% alcohol for preservation. Helminth identification was carried out on the basis of morphological and morphometric characteristics, with the help of identification keys from Vicente [11] and Gibson et al. [21]. The mean intensity and mean abundance were calculated according to Bush et al. [22].

## 3. Results

### 3.1. Tick Collection and Rickettsial Infection

**FIGURE 2:**
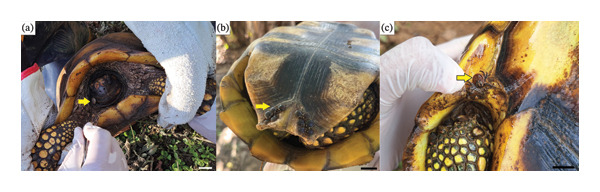
Ticks (yellow arrows), *Amblyomma humerale*, found on *Chelonoidis denticulatus* at the Rio Acre Ecological Station, Western Amazonia, Brazil. Scale bar = 2 cm.

All seven samples of *C. denticulatus* collected were infested with ticks (100% occurrence). A total of 51 samples of *A. humerale* (Figure 2) were collected from various body regions, with a mean intensity of 7.28. A total of 40 males (78.5%) and 11 females (21.5%) were included (Tables 1 and 2).

**TABLE 1:** Number of *Amblyomma humerale* individuals parasitizing *Chelonoidis denticulatus*, according to sampling date, capture method, and tick sex, at the Rio Acre Ecological Station in Assis Brasil, Acre, Brazilian Western Amazon (from February 3 to February 10, 2024).

Collection date	Capture method	Sex	Number of ticks		Total
			Male	Female	
February 3, 2024	Occasional encounter	Male	8	1	9
February 3, 2024	Occasional encounter	Male	7	3	10
February 5, 2024	Occasional encounter	Female	3	0	3
February 6, 2024	Active search	Male	2	2	4
February 6, 2024	Active search	Male	6	3	9
February 7, 2024	Active search	Male	3	1	4
February 10, 2024	Trap	Female	11	1	12
Total			40	11	51

**TABLE 2:** Locations of *Amblyomma humerale* individuals found parasitizing *Chelonoidis denticulatus* according to region and quantity per capture at the Rio Acre Ecological Station in Assis Brasil, Acre, Brazilian Western Amazon (from February 3 to February 10, 2024).

Capture region	Quantity by region
Left front limb	1
Right front limb	17
Left hind limb	3
Right hind limb	1
Carapace	24
Plastron	3
Head	2

A total of 51 *A. humerale* adults were collected parasitizing *C. denticulatus* during the sampling period (Table 1). Most ticks were obtained through occasional encounters on February 3 (*n* = 19) and through active searches on February 6 (*n* = 13). Females were less abundant (*n* = 11) compared to males (*n* = 40), but both sexes were recorded across different dates and capture methods, indicating active parasitism throughout the week of sampling. The spatial distribution of ticks on the host’s body (Table 2) revealed a preference for the carapace (*n* = 24) and the right front limb (*n* = 17), which together accounted for over 80% of all attachment sites. Other regions such as the plastron (*n* = 3), left hind limb (*n* = 3), head (*n* = 2), left front limb (*n* = 1), and right hind limb (*n* = 1) had fewer ticks. This distribution suggests that adults of *A. humerale* exhibit nonrandom attachment behavior, concentrating on exposed or structurally favorable regions of the host.

*A. humerale* were tested for *Rickettsia* infection. However, none of the ticks tested positive for *Rickettsia* agents.

### 3.2. Coproparasitological Identification

Eggs of Oxyurida (*n* = 3, 50%), Ascaridida (*n* = 2, 33.33%), Cosmocercoidea (*n* = 5, 83.33%), and Strongylida (*n* = 2, 33.33%) were identified, as were cysts of *Entamoeba* spp. (*n* = 2, 33.34%), oocysts of Coccidia (*n* = 1, 16.67%), and trophozoites of *Balantidium* spp. (*n* = 1, 16.67%).

### 3.3. Helminth Collection From Viscera

Overall, three different parasites were identified: *L. zschokkei* (6/10), *C. variabilis* (6/10), and *Haltrema* spp. (1/10) (Figures 3, 4, and 5). In total, six (60.0%) animals were parasitized by at least one of the three species detected in this study, resulting in the recovery of a total of 45,774 helminths. The mean intensity of parasitism was as follows: *L. zschokkei* 7208.3, *C. variabilis* 416.6, and *Haltrema* spp. 24. The abundances were *L. zschokkei* 4325.0, *C. variabilis* 250.0, and *Haltrema* spp. 2.4. Morphometric analyses were performed on 10 parasites of each species, following parameters in the literature and adding additional characteristics for the species (Tables 3 and 4). Specimens were deposited in the Helminthological Collection of the Federal University of Jataí: CHUFJ‐0045 (*C. variabilis*), CHUFJ‐0046 (*L. zschokkei*), and CHUFJ‐0047 (*Haltrema* spp.).

FIGURE 3
*Labiduris zschokkei* in *Chelonoidis denticulatus* from Western Amazonia. (a) Anterior end. rn = ring nerve; ae = anterior esophagus; i = isthmus; and b = esophageal bulb. (b) Female posterior end (ventral view). an = anus. (c) and (d) Male posterior end (lateral view). sp = spicules; t = tail; ca = caudal appendix; pcp = precloacal papillae; pap = post anal papillae.

**Figure:**
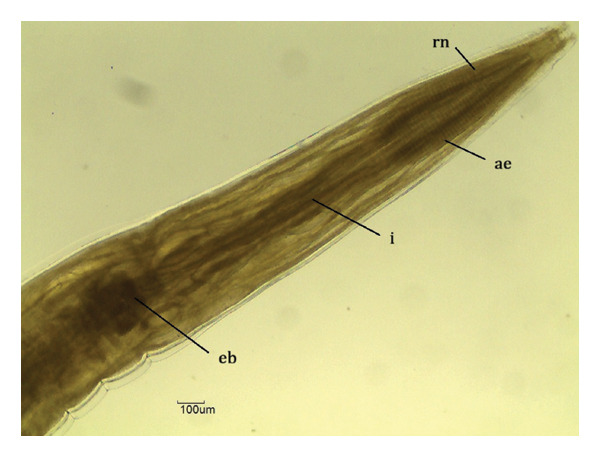
(a)

**Figure:**
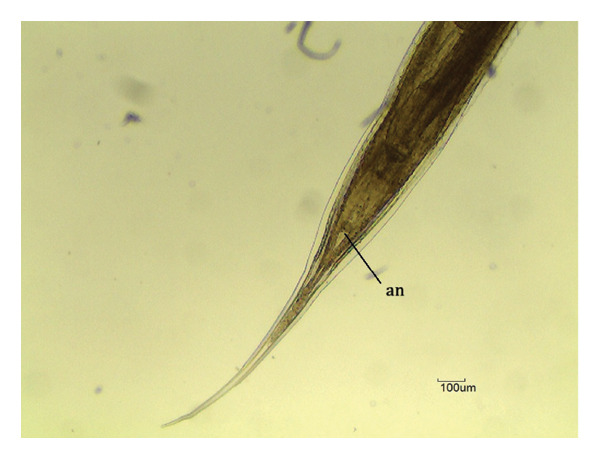
(b)

**Figure:**
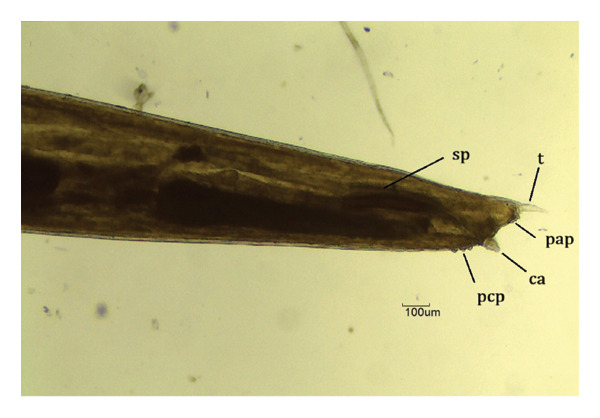
(c)

**Figure:**
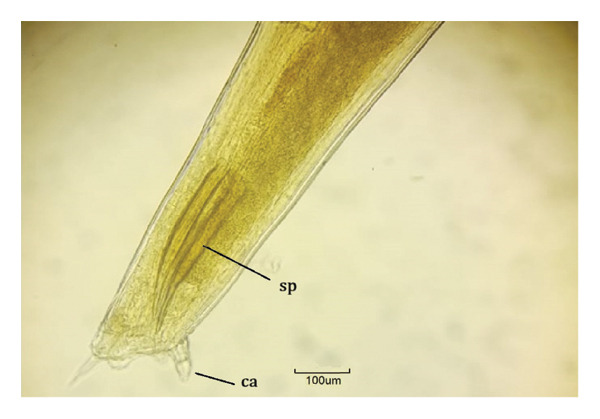
(d)

FIGURE 4
*Chapiniella variabilis* in *Chelonoidis denticulatus* from Western Amazonia. (a) Anterior end. nl = head necklace with crown; e = esophagus; nr = nerve ring. (b) Male posterior end (ventral view). sp = spicules; g = gubernaculum; cb = copulatory bursa. (c) Female posterior end (lateral view). an = anus; v = vulva.

**Figure:**
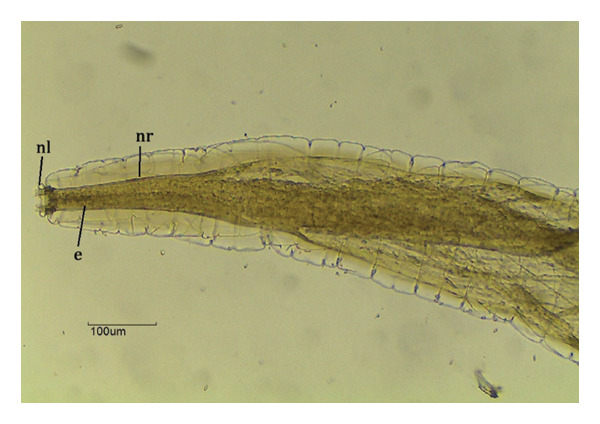
(a)

**Figure:**
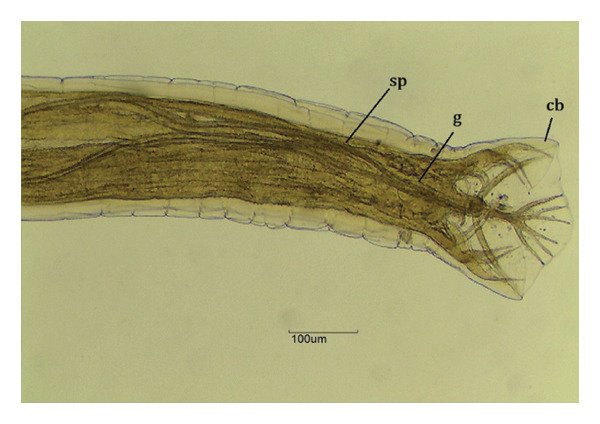
(b)

**Figure:**
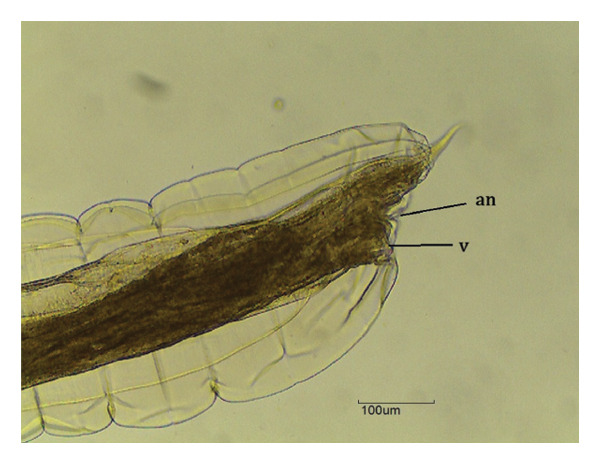
(c)

FIGURE 5
*Haltrema* spp. in *Chelonoidis denticulatus* from Western Amazonia. (a) Light micrograph. (b) Morphological diagram. os = oral sucker; oc = oral cavity; c = cecum; at = anterior testis; pt = posterior testis; vg = vitelline gland; ov = ovary; ac = acetabulum. Scale bar = 1000 μm.

**Figure:**
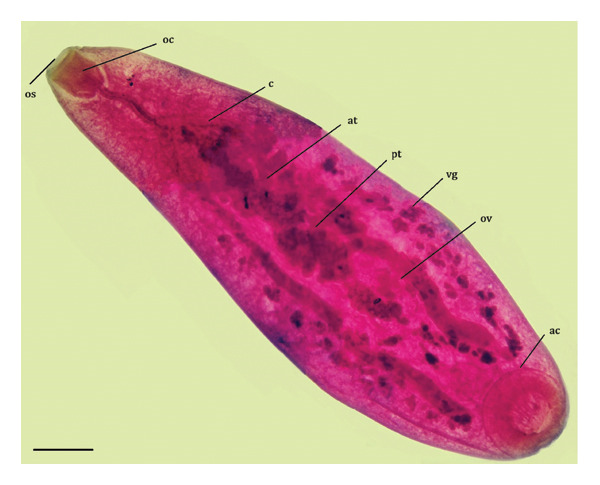
(a)

**Figure:**
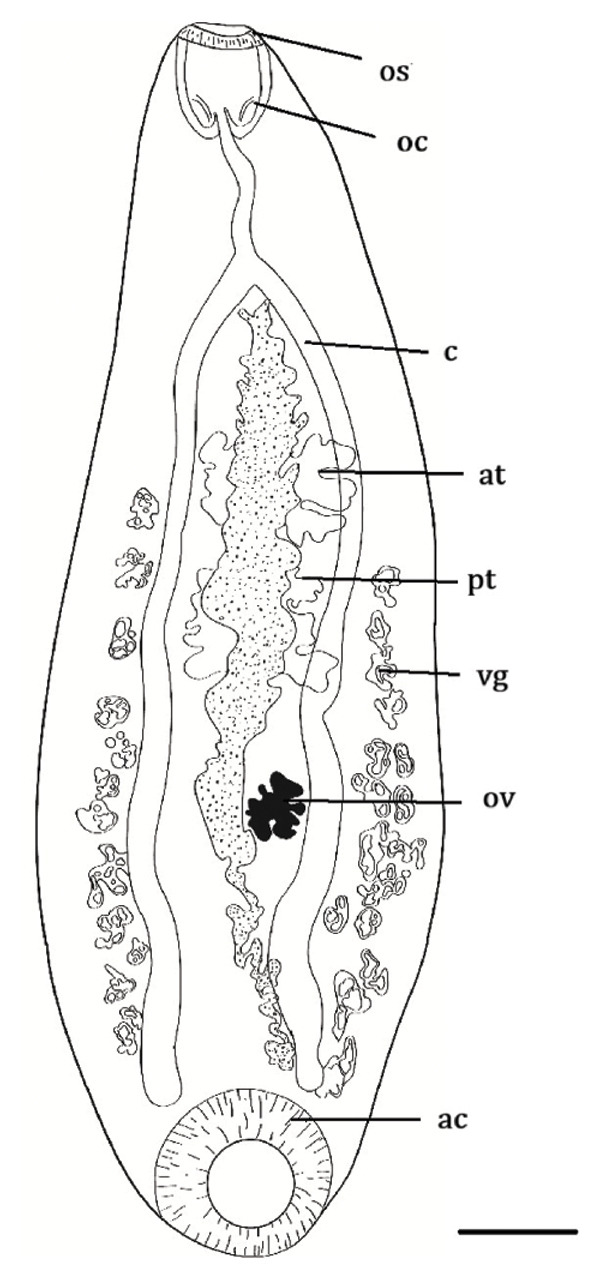
(b)

**TABLE 3:** Morphological and morphometric data (μm) of *Labiduris zschokkei and Chapiniella variabilis found in Chelonoidis denticulatus* consumed by the human population in Cruzeiro do Sul.

Characteristics	*Labiduris zschokkei*		*Chapiniella variabilis*	
	Male	Female	Male	Female
Total length	15,159.06–2212.26	22,120.26–1625.03	10,592.9–601.80	13,117.91–904.35
Width	820.24–147.24	912.25–179.29	421.58–43.90	453.74–46.75
Buccal capsule	—	—	136.19–13.16	129.77‐26.79
Esophagus	2572.5–480.64	2764.57–380.35	595.11–58.74	631.18–63.58
Excretory pore	1669.91–438.14	1937.41–699.25	544.75–111.55	673.04–219.07
Nerve ring to anterior end	107.9–21.74	173.28–46.48	147.35–35.88	155.99–23.23
Anterior bulb	183.31–24.16	201.18–74.02	—	—
Posterior bulb	417.69–66.75	407.64–88.82	—	—
Gubernaculum	187.8–66.19	—	—	—
Spicules	—	—	2578.85–422.24	—
Right spicules	933.98–112.35	—	—	—
Left spicules	924.32–108.79	—	—	—
Cervical papillae	—	—	623.89–171.26	704.47–61.95

*Note:* Mean (M) and standard deviation (SD) of the samples.

**TABLE 4:** Morphological and morphometric data (μm) of *Haltrema* spp. found in *Chelonoidis denticulatus* consumed by the human population in Cruzeiro do Sul.

Variable	Mean–standard deviation
Total length	8750–680
Total width	3650–410
Oral sucker	493.92–56.16
Oral cavity	667.45–22.96
Acetabulum length	1076.3–102.76
Acetabulum width	1283.02–46.04
Eggs	109.41–6.25
Ovary length	402.51–83.47
Ovary width	361.91–63.28
Anterior testis length	821.52–143.68
Anterior testis width	913.7–179.05
Posterior testis length	861.45–222.62
Posterior testis width	793.36–178.24
Vitelline gland distribution	4085.8–559.0

## 4. Discussion

In this study, infestation by *A. humerale* on *C. denticulatus* exhibited a clear pattern, with most individuals located on the carapace and the right front limb. This nonrandom distribution suggests that these regions may offer structural protection and easier access for tick attachment, especially under natural conditions. Additionally, the noticeable predominance of males over females and their presence across different capture dates and methods reinforce the role of *C. denticulatus* as an important and consistent host for the adult stages of *A. humerale* in the southwestern Brazilian Amazon. These infestation patterns, together with the concentration of ticks in aggregated clusters, align with the expected ecological behavior observed in adult *Amblyomma* species.

Parasitism by *A. humerale* has generally been associated with terrestrial turtle species [8]. In Brazil, literature records indicate that in tortoises, especially studies with *C. denticulatus*, there are no reports of other tick species found on these animals. However, *Amblyomma* (*Cernyomma*) *extraculatum* was reported on *C. denticulatus* in Peru [23]. The tick specimens identified in this research are consistent with data previously described in the literature [6–9, 24, 25], with a higher incidence of encounters on the carapace of males, particularly in groups, than on that of females. The males remain attached during feeding and mating, whereas females leave their hosts to lay eggs. This fact can be explained by the greater need for males to spend more time feeding on blood [26].

In a study investigating tick infestations in reptiles and amphibians captured in Central Amazonia and evaluating the presence of rickettsiae, only one *A. humerale* nymph collected from a common tegu tested positive for the *gltA* gene, showing 100% identity with an *R. amblyommatis* sequence from Brazil (GenBank accession number: KY273595.1) [5]. Conservation units are recognized as key indicators in biodiversity conservation strategies, yet few *Rickettsia* species have been described in animals residing within these conservation units [27]. In our study, no DNA from rickettsial agents was detected, which is considered normal and demonstrates that ticks in this host group actually have a low prevalence rate of rickettsiae.

Through fecal examinations, we observed the presence of protozoan cysts, such as *Entamoeba* spp., which are primarily indicative of environmental contamination and represent a potential zoonotic risk [28]. However, none of the helminths presented records of species shared between humans and tortoises. Helminth eggs were relatively well distributed, with the exception of the Cosmocercoidea family, which was parasitized in 83.33% of the animals and may be linked to the occurrence of *Labiduris* spp. reported from the viscera analysis; this family is the most abundant parasite. Structures compatible with Strongylida eggs, which may be related to *C. variabilis*, were also described in the analyses of the viscera of animals consumed in the municipality of Cruzeiro do Sul, Acre, in this study.

*C. variabilis* is a strongyloid nematode from the superfamily Strongyloidea and has been reported to parasitize the intestinal region of *C. denticulatus* in Pará and Piauí [29, 30]. Its main distinguishing features from those of the other five species are the 18‐element leaf crowns at the cephalic end, the proportion between the length of the nerve ring and the anterior end divided by the length of the esophagus, the ratio between the length of the spicule and the length of the body, and the diameter of the oral capsule. These parameters help reinforce the identification of the species of the samples collected in this study [31–34]. The genus *Labiduris*, which parasitizes *C. denticulatus*, such as *L. gulosa* in Pará and *L. irineuta* in Rio de Janeiro, has been recorded in Brazil, and the occurrence of *L. zschokkei* parasitizing *C. denticulatus* in the intestinal region has also been reported [11].

The helminth fauna of *C. denticulatus* in the western Amazon was predominantly composed of nematode helminths, which presented greater parasitism intensity than the single trematode species found, namely, *Haltrema* spp., which belong to the superfamily Paramphistomatidae and the family Cladorchiidae [35]. Although a slight alteration in the description of the samples was found in this study, the key matches the genus, but it does not allow for species‐level identification [36, 37]. The occurrence of the genus *Haltrema* in *C. denticulatus* specimens in Acre increases the records of this parasite after a 59‐year hiatus without data and its occurrence in another state of Brazil, within the Neotropical region of South America. The only previous known report of this genus parasitizing *C. denticulatus* was that of *H. avitellina* adults in the stomachs of *C. denticulatus* specimens in Pará [36]. However, *H. avitellina* has also been described in the stomachs of *Podocnemis expansa* in Tocantins [38] and Pará [39]. Other records of occurrence in South America are found in Peru [40] and Venezuela, specifically in *P. expansa* and *Podocnemis unifilis* [39, 41].

## 5. Conclusion

This study provides baseline data on the parasite fauna of *C. denticulatus* in the far western Brazilian Amazon, a region where parasitological surveys in reptiles remain scarce despite intense human and wildlife interaction. Although none of the helminths detected are known to be zoonotic and all tick samples were negative for *Rickettsia* spp., the occurrence of *Entamoeba* cysts highlights the potential for environmental contamination and underscores the importance of continuous monitoring. By expanding the records of trematodes and nematodes associated with this species and documenting the spatial distribution of *A. humerale* on tortoises, our findings contribute to the ecological and sanitary understanding of wildlife in the region and reinforce the need for long‐term surveillance programs rather.

## Author Contributions

Conceptualization, Ester Nascimento da Costa, Dirceu Guilherme de Souza Ramos, and Francisco Glauco de Araújo Santos; methodology, Ester Nascimento da Costa, Caio Bonfanti Gomes, Rayná Girard Madeira, João José de Souza Moura, Ana Paula Carvalho Gomes, Iago de Sá Moraes, and Victória Luiza de Barros Silva; validation, Richard de Campos Pacheco, Dirceu Guilherme de Souza Ramos,, and Francisco Glauco de Araújo Santos; formal analysis, Ester Nascimento da Costa, João José de Souza Moura, Victória Luiza de Barros Silva, Iago de Sá Moraes, Reiner Silveira de Morae, and Dirceu Guilherme de Souza Ramos; investigation, Ester Nascimento da Costa, Caio Bonfanti Gomes, Rayná Girard Madeira, and Muriele Furtado de Assis; resources, Muriele Furtado de Assis, Richard de Campos Pacheco, Dirceu Guilherme de Souza Ramos, and Francisco Glauco de Araújo Santos; writing–original draft preparation, Ester Nascimento da Costa, Caio Bonfanti Gomes, Rayná Girard Madeira, João José de Souza Moura, Muriele Furtado de Assis, Victória Luiza de Barros Silva, Ana Paula Carvalho Gomes, and Iago de Sá Moraes; writing–review and editing, Reiner Silveira de Morae, Richard de Campos Pacheco, Dirceu Guilherme de Souza Ramos, and Francisco Glauco de Araújo Santos; supervision, Dirceu Guilherme de Souza Ramos and Francisco Glauco de Araújo Santos; project administration, Dirceu Guilherme de Souza Ramos and Francisco Glauco de Araújo Santos; funding acquisition, Richard de Campos Pacheco, Dirceu Guilherme de Souza Ramos, and Francisco Glauco de Araújo Santos.

## Funding

The study was funded by Fundação de Amparo à Pesquisa do Estado de Goiás (FAPEG) (grant nos. 202310267001408 and 202510267000577); by Conselho Nacional de Desenvolvimento Científico e Tecnológico (CNPq) with research productivity grant (Richard de Campos Pacheco and Dirceu Guilherme de Souza Ramos); and by Coordenação de Aperfeiçoamento de Pessoal de Nível Superior (CAPES) for scholarship (Ester Nascimento da Costa, Ana Paula Carvalho Gomes, Victória Luiza de Barros Silva, and Iago de Sá Moraes).

## Conflicts of Interest

The authors declare no conflicts of interest.

## Data Availability

Data sharing is not applicable to this article as no datasets were generated or analyzed during the current study.
